# Concurrent Aerobic and Resistance Training Has Anti-Inflammatory Effects and Increases Both Plasma and Leukocyte Levels of IGF-1 in Late Middle-Aged Type 2 Diabetic Patients

**DOI:** 10.1155/2017/3937842

**Published:** 2017-06-21

**Authors:** Giosuè Annibalini, Francesco Lucertini, Deborah Agostini, Luciana Vallorani, Annamaria Gioacchini, Elena Barbieri, Michele Guescini, Lucia Casadei, Annunziata Passalia, Marta Del Sal, Giovanni Piccoli, Mauro Andreani, Ario Federici, Vilberto Stocchi

**Affiliations:** ^1^Department of Biomolecular Sciences, Division of Exercise and Health Sciences, University of Urbino Carlo Bo, Urbino, Italy; ^2^Department of Diabetology and Endocrinology, Asur Marche Area Vasta 1, S. Maria della Misericordia Hospital, Urbino, Italy

## Abstract

Type 2 diabetes (T2D) is an age-related chronic disease associated with metabolic dysregulation, chronic inflammation, and activation of peripheral blood mononuclear cells (PBMC). The aim of this study was to assess the effects of a concurrent exercise training program on inflammatory status and metabolic parameters of T2D patients. Sixteen male patients (age range 55–70) were randomly assigned to an intervention group (*n* = 8), which underwent a concurrent aerobic and resistance training program (3 times a week; 16 weeks), or to a control group, which followed physicians' usual diabetes care advices. Training intervention significantly improved patients' body composition, blood pressure, total cholesterol, and overall fitness level. After training, plasma levels of adipokines leptin (−33.9%) and RBP4 (−21.3%), and proinflammatory markers IL-6 (−25.3%), TNF-*α* (−19.8%) and MCP-1 (−15.3%) decreased, whereas anabolic hormone IGF-1 level increased (+16.4%). All improvements were significantly greater than those of control patients. Plasma proteomic profile of exercised patients showed a reduction of immunoglobulin K light chain and fibrinogen as well. Training also induced a modulation of *IL-6*, *IGF-1*, and *IGFBP-3* mRNAs in the PBMCs. These findings confirm that concurrent aerobic and resistance training improves T2D-related metabolic abnormalities and has the potential to reduce the deleterious health effects of diabetes-related inflammation.

## 1. Introduction

Type 2 diabetes (T2D) is an age-related chronic disease associated with a reduction in skeletal muscle strength and mass and an increase in body fatness and blood inflammatory markers [1]. The reduction of muscle mass leads to impaired physical function, high risk of falls and fractures, and worsening of glycemic control, since skeletal muscle mass represents the largest insulin-sensitive tissue of the body [2]. Moreover, the concomitant increase of fat mass is directly associated with insulin resistance and chronic inflammation in T2D patients [1, 3]. Indeed, adipose tissue is an active endocrine tissue that secretes adipokines, such as adiponectin, leptin, and retinol-binding protein 4 (RBP4), regulating glucose homeostasis as well as inflammatory responses [4, 5]. Notably, subclinical inflammation also exacerbates overall risk of T2D-related complications, such as cardiovascular disease [6]. In T2D patients, a chronic increase in plasma levels of acute phase proteins, such as C-reactive protein (CRP) and fibrinogen, has been observed in both cross-sectional and prospective studies [1]. Serum concentrations of cytokines and chemokines, such as interleukin 6 (IL-6), tumor necrosis factor-*α* (TNF-*α*), and monocyte chemoattractant protein-1 (MCP-1), have been also reported to be slightly increased in subjects with T2D [3]. Conversely, a low concentration of anabolic signals, such as serum level of insulin-like growth factor-1 (IGF-1), has been found in T2D patients [7]. A chronically elevated level of inflammatory markers is also associated with the activation of immune cells, such as peripheral blood mononuclear cells (PBMC). Indeed, leukocytes isolated from subjects with poorly controlled diabetes have shown an increased expression of the inflammatory markers and an increased ability to adhere to the endothelium [8, 9].

Both physical activity and exercise training have been proposed as natural antioxidant and anti-inflammatory strategies to prevent the evolution and complication of T2D, decreasing plasma levels of inflammatory markers and increasing the activity of the IGF-1 axis in T2D patients [10–12]. However, literature shows conflicting results about the anti-inflammatory proprieties of exercise training, maybe attributable to different training regimens [10, 13–15]. For example, Oberbach et al. [16] reported a reduction in the plasma IL-6 levels after an aerobic exercise training program while Jorge et al. [17] did not. Recently, the results obtained from the Italian Diabetes and Exercise Study suggested that the anti-inflammatory effect of exercise training was dependent on type, dose, and intensity of exercise [10]. In particular, T2D patients performing supervised sessions of aerobic plus resistance exercise (i.e., concurrent training) showed a higher reduction of inflammatory markers compared to patients performing aerobic training alone, despite similar caloric expenditure [10]. Accordingly, a meta-analysis that examined the effects of different modes of exercise training (aerobic, resistance, or concurrent training) on related risk factors for complications of diabetes showed that concurrent training was generally superior to aerobic or resistance training alone [18].

It is widely accepted that the physiological adaptations to resistance training differ from those of aerobic training [19, 20]. Indeed, the current paradigm is to combine endurance and strength training to optimize health and general cardiovascular benefits in T2D patients. In fact, both aerobic and resistance training are currently recommended by the American College of Sports Medicine (ACSM) and the American Diabetes Association (ADA) for diabetes management [21, 22].

However, the effects on inflammation of a concurrent aerobic and resistance training intervention prescribed according to the ACSM-ADA guidelines [21–23] have yet to be addressed properly. Therefore, the aim of the present study was to evaluate the effectiveness of concurrent training in reversing inflammation in late middle-aged patients with T2D. We hypothesized that concurrent training might improve physical fitness parameters and body composition of T2D patients, hence decreasing inflammation associated with T2D.

## 2. Materials and Methods

### 2.1. Participants

Sixteen 55 to 70 year-old male subjects with T2D (defined according to the ADA criteria [24]) without known cardiovascular disease were enrolled. Additional requirements consisted in diabetes duration >2 years, body mass index (BMI) between 25 and 30 kg/m^2^, ability to walk without physical assistance, and eligibility after cardiovascular evaluation. Prior physical activity habits were evaluated using the 7-day Physical Activity Recall questionnaire [25], and volunteers were deemed sedentary if their leisure time physical activity energy expenditure was lower than 300 kcal/d.

All subjects reported that their body weight had been stable for at least three months prior to the study. All participants received standard diabetes care including dietary prescription and pharmacological treatment (hypoglycemic agents), but none was treated with insulin or thiazolidinedione. Since medications for treatment of hypertension and dyslipidemia have also demonstrated anti-inflammatory effects, T2D patients taking antihypertensive or lipid-lowering medications were excluded [26]. Subjects were counseled to maintain dietary intake unchanged throughout the study. To this purpose, dietary habits were assessed with 24-hour dietary recall interviews performed at baseline, at week 8, and at the end of the study. Participants were also advised to not change their medications during the study. This investigation was approved by the Human Ethics Committee of the local university, and all subjects signed an informed consent form before enrollment.

### 2.2. Experimental Design

Participants were randomly assigned to either an intervention group (EXE), which underwent a 16-week concurrent aerobic and resistance training program (see Section 2.3), or a control group (CON), which continue the usual diabetes management routine during the 16-week experimental period. Before and after the trial, the participants of both groups underwent the assessments described in Sections 2.4–2.9.

### 2.3. Exercise Training Program

The exercise program consisted in a concurrent aerobic and resistance training regimen. The proportion of aerobic and resistance exercise modalities within each training session was not established a priori since exercise was prescribed according to the joint recommendations of ACSM and ADA and following the current ACSM's practical guidance [21–23]. Accordingly, the proportion of the two exercise modalities changed throughout the concurrent training intervention since a rate of progression for duration and intensity increments was adopted as well (see below).

Patients were scheduled to exercise in the afternoon (2:00 p.m.) in the absorptive state (2 hours after lunch) on 3 nonconsecutive days of each week.

In order to match the recommendations of the weekly frequency of resistance training (2-3 times per week), aerobic and resistance training was completed within the same training session [21, 22]. Aerobic exercise was always carried out before resistance in order to reduce to the minimum the injury risks associated with resistance exercise performed without proper warm-up. Endurance exercise was performed by walking on a treadmill with a gradual and progressive increase in both intensity (40% to 65% of heart rate (HR) reserve) and duration (30 to 60 minutes) throughout the training intervention. Aerobic exercise duration increases (about 5 to 10 minutes according to participant's responses) were privileged over the first 2 weeks, while duration and intensity (about 10% of HR reserve every 4 weeks) were increased alternatively in the following 14 weeks. Strength exercises were performed on the horizontal leg press, on the lat pull-down, on the lat machine, and on the chest press and gradually increased from 2 to 4 sets of 20 to 12 repetitions on each isotonic machine, with loads ranging from 40% to 60% of 1-repetition maximum (1-RM). Strength exercise progression followed the progressive overload principle by alternating increments of the resistance (about 10% of 1-RM in 4 weeks) with increments of the number of sets (1 more set in 8 weeks).

### 2.4. Exercise Testing

Aerobic and muscular fitness were assessed on two separate occasions, with a 2-day recovery period in-between, in accordance with the testing guidelines of the ACSM [27]. Before starting the first testing session, resting HR and blood pressure were measured after 10-minute rest with the participant sitting on a chair.

#### 2.4.1. Aerobic Fitness

Maximal oxygen consumption (VO_2max_) was estimated on a treadmill (Panatta Sport, Apiro, MC, Italy) by means of a submaximal graded incremental walking test (modified Balke protocol [27]). The test was terminated when the HR of the participant approached the 85% of the age-predicted maximal HR (HR_max_; calculated according to Gellish et al. [28]). A linear regression was calculated over the observations of HR (recorded at the end of each 3 min stage performed) and the relevant treadmill grades and used to estimate the exercise intensity at the HR_max_, which was converted into estimated VO_2max_ according to a published equation for walking [27].

#### 2.4.2. Muscular Fitness

Both muscular strength (1-RM) and endurance (multiple RM; m-RM) were evaluated using the leg-press, the chest-press, and the lat pull-down machines (Panatta Sport, Apiro, MC, Italy).

The 1-RM was estimated according to the regression equation of Brzycki [29]. Briefly, following a warm-up of 2 sets of 15–20 repetitions with approximately the load corresponding to 50% of the expected 1-RM, the participant performed as much repetitions as possible, until muscular fatigue occurred, with a load that he was able to lift between 6 and 10 times. The number of repetitions performed and the load selected was then computed in the regression equation [29] and estimated 1-RM retained for subsequent analysis.

The m-RM was assessed as the maximum number of repetitions performed on each exercise with a load corresponding to 40% of the estimated 1-RM. The imposed rhythm for the repetitions was set at 60 beats per minute (a metronome was used), and when it was not met for 2 consecutive reps and/or correct technique was no more maintained, the test was terminated and the number of reps performed retained for subsequent analysis.

### 2.5. Anthropometry and Body Composition

Height (head in the Frankfurt plane) and weight (light clothes, without shoes) were measured with the patient standing, and BMI was calculated (kg/m^2^). Waist and hip circumferences were assessed using standard methods (average of triplicate measures), while body composition (expresses as fat and fat-free percentages of body weight) was assessed by dual-energy X-ray absorptiometry (DXA LUNAR® GE Healthcare, Milan, Italy).

### 2.6. Biochemical Analyses

Blood samples were taken following an overnight fast and at least 72 h after the final training session. Concentrations of glycated hemoglobin (HbA1c), glucose, serum triglycerides, total and HDL cholesterol, and creatininemia were determined using routine laboratory methods in the local clinic by an auto-analyzer (Beckman Coulter, Milan, Italy). Leptin, adiponectin, RBP4, IL-6, MCP-1, TNF-*α*, high sensitivity CRP (hs-CRP), IGF-1, and IGF binding protein 3 (IGFBP-3) were measured using R&D Systems Quantikine ELISA kits (Space Import-Export S.r.l., Milan, Italy). All samples were assayed in duplicate. The intra-assay coefficients of variation for cytokine assays ranged from 2.5% to 8.5%.

### 2.7. PBMC Isolation, RNA Extraction, and cDNA Synthesis

PBMCs were isolated from EDTA blood by density gradient centrifugation on Lymphoprep™ by Axis-Shield (Sentinel Diagnostic, Milan, Italy). Total RNA was extracted and purified using the Omega Bio-Tek E.Z.N.A.™ total RNA kit (VWR International, Milan, Italy) according to the manufacturer's instructions. After DNA digestion with DNase I enzyme (Qiagen, Milan, Italy), complementary DNA was synthesized from 1 *μ*g of total RNA using Omniscript RT (Qiagen, Milan, Italy) and random hexamers.

### 2.8. Real-Time RT-PCR Quantification

PCR was performed with two microliters of cDNA in a Bio-Rad iCycler iQ Multi-Color Real-Time PCR Detection System using 2x Quantitect SYBR PCR kit (Qiagen, Milan, Italy) and 300 nM of each primer. The amount of the target transcripts was normalized to the reference gene glyceraldehyde-3-phosphate dehydrogenase (*GAPDH*). Gene expression data are expressed using the 2^–ΔCT^ method as described by Pfaffl [30]. The real-time PCR conditions were 95°C for 10 min and followed by 40 cycles of three steps at 95°C for 30 sec, 60°C for 30 sec, and 72°C for 30 sec. The specificity of the amplification products was confirmed by examining thermal denaturation plots and by sample separation in a 4% DNA agarose gel. The sequences of primers used in real time RT-PCR quantification are listed in Supplementary Table S1 available online at https://doi.org/10.1155/2017/3937842.

### 2.9. Plasma Proteome Analysis

Two pools of plasma samples were obtained by mixing equal proportions of plasma [31], from the eight diabetic subjects of the EXE group, collected before and after the completion of the training program, and prefractioned using the ProteoMiner™ kit Large-Capacity Kit (Bio-Rad Laboratories, Hercules, CA, USA) according to the manufacturer's protocol. Proteins elution was carried out with urea lysis buffer (8 M urea, 4% CHAPS, 65 mM DTE, and 40 mM Tris base). The protein concentration of each sample was determined according to Bradford [32]. Two-dimensional electrophoresis (2-DE) was carried out as previously described [33]. Analytical gels were stained with silver nitrate [34]. Semipreparative gels for mass spectrometry analysis were stained with Brilliant Blue R-250 (Sigma-Aldrich, Milan, Italy) according to the manufacturer's procedure. Gel images were acquired by the Fluor-S MAX multi-imaging system (Bio-Rad Laboratories Italy, Milan, Italy), and the data were analyzed with the ImageMaster 2D Platinum software. Method for in gel digestion was adapted from Shevchenko et al. [35] as previously described [36]. LC-ESI-MS/MS analysis was performed using a Q-TOF microTM mass spectrometer (Micromass, Manchester, UK) as previously described [37].

### 2.10. Statistical Analysis

Data analyses were performed using SPSS statistical software (v13.0). Baseline value differences between groups were assessed with the Student *t*-test for unpaired data. Percent changes from baseline between the 2 groups (((value at week 16−baseline value)/baseline value) ^∗^ 100) were compared with multiple analysis of covariance (ANCOVA), adjusting for baseline values as covariates. A *p* value <0.05 was considered statistically significant.

## 3. Results

Participants' baseline and postintervention parameters are shown in Table 1. There were no significant differences between groups at baseline (all *p* > 0.5). All subjects of the EXE group completed the 16-week training program, and no major adverse effects or health problems attributable to the assessments or to the training sessions were recorded.

### 3.1. Anthropometry, Body Composition, Fitness Level, and Clinical Parameters

Compared to the CON group (Table 1), the EXE group significantly improved body weight, BMI, waist and hip circumferences, body fat and fat-free mass, aerobic and muscular fitness, and systolic and diastolic resting blood pressure. Total cholesterol improvement was significantly greater in the EXE group over the CON group, while LDL and HDL cholesterol, blood glucose, HbA1c, and creatininemia did not change between the two groups after the trial.

### 3.2. Plasma Adipokines, Inflammatory Markers, and Anabolic Hormone Concentrations

A significantly greater reduction was observed for the adipokines leptin and RBP4 in the EXE group compared to the CON group. Adiponectin level did not change between the two groups, as well as hs-CRP and IGFBP-3. Compared to the CON group, plasma level of the proinflammatory markers IL-6, MCP-1, and TNF-*α* decreased and IGF-1 increased to a greater extent in the EXE group (Table 2).

### 3.3. Plasma Proteome

**Figure 1:**
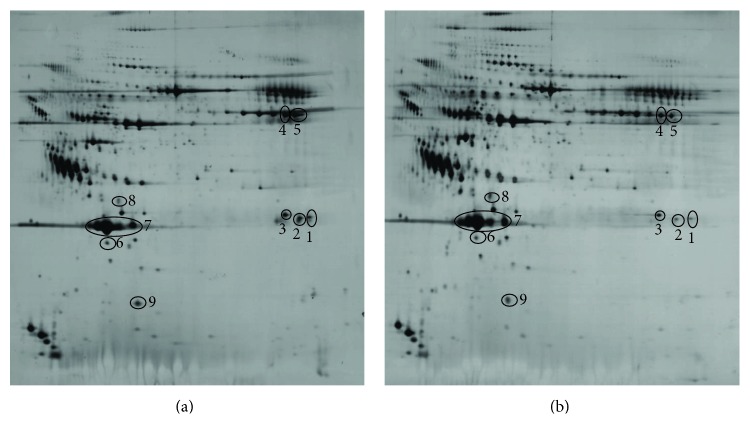
2-DE maps of plasma proteins from EXE group before (a) and after (b) the 16-week exercise training program. Differentially expressed spots are indicated by black circles and numbered according to Table 3.

Figure 1 shows the 2-DE maps of plasma proteins obtained at baseline (a) and after training (b). The image analysis allowed the detection of proteins whose expression varied at least 2-fold between the two conditions, 9 of which were identified by mass spectrometry (Table 3).

The level of K light chain of immunoglobulin (Figure 1, spots 1, 2, and 3), fibrinogen (Figure 1, spots 4 and 5), RBP4 (Figure 1, spot 6), and transthyretin (TTR) (Figure 1, spot 9) decreased after the training program while apolipoproteins (Apo) A1 and ApoE (Figure 1, spots 7 and 8) increased.

### 3.4. PBMC Gene Expression Levels

**Figure 2:**
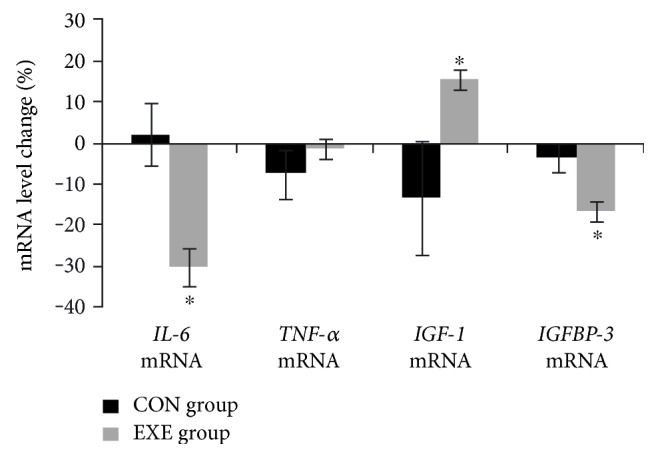
mRNA expression of *IL-6*, *TNF-α*, *IGF-1*, and *IGFBP-3* in peripheral monocytes of control (CON) and exercise (EXE) groups at baseline (white bars) and after the 16-week exercise training program (black bars). Values are means ± SD. ∗Significant between-group differences, as assessed by ANCOVA (baseline values as covariates).

The mRNA expression of *IL-6* and *IGFBP-3* decreased in the PBMC of the EXE group while the *IGF-1* mRNA showed an opposite trend with a statistically significant difference (Figure 2). The *TNF-α* mRNA did not change significantly after the trial.

## 4. Discussion

The role of inflammation in the pathogenesis of T2D and associated complications is now well established [1]; conversely, the anti-inflammatory effect of exercise training is still under debate [10, 13–15, 38].

The present study analyzed the physiological and immunological responses of late middle-aged T2D patients to a 16-week concurrent aerobic and resistance training intervention prescribed according to the recommendations of the ACSM and ADA [21–23]. We found that the concurrent exercise training yields significant beneficial effects on body composition and fitness level, blood pressure, inflammatory mediators, growth factors, and appetite-regulating hormones. These findings have clinical implications given the high prevalence of T2D among the people aged 55–70 [39].

The training-induced reduction of fat mass of the EXE group is particularly important since several studies demonstrated that adipose tissue is an active endocrine tissue and that, in T2D patients, an excess of adipose tissue is accompanied by infiltration of immune cells, increased release of adipokines and development of systemic low-grade inflammation [1, 3]. Hence, the training-induced reduction of adipokines leptin and RBP4 reported in this study was likely related to the reduction of fat mass. It is worthy of note that also the proteomic analysis of plasma from the EXE group showed a decrease of the RBP4 and TTR, which is a protein that plays a critical role in stabilizing RBP4 in the circulation [40]. Adipokines are central to the dynamic control of energy metabolism, communicating the nutrient status of the organism to the tissue responsible for controlling energy intake and expenditure and insulin sensitivity [4, 41]. Additionally, several studies have linked adipokines with the pathogenesis of obesity-related inflammation [42]. For instance, a recent study conducted on obese mice showed that lowering RBP4 level by disrupting TTR was effective in improving insulin resistance and adipose tissue inflammation [40]. In our study, the training-induced reductions of leptin, RBP4, and TTR were associated with favorable changes of inflammatory markers in the EXE group. In particular, we found a reduction of several proinflammatory cytokines (IL-6, MCP-1, and TNF-*α*), an increase of IGF-1, and a modulation of *IL-6, IGF-1*, and *IGFBP-3* mRNAs in the PBMC isolated from the EXE group. Although circulating inflammatory cytokines originate from multiple sources [1, 3, 6], we may speculate that the anti-inflammatory effect of the exercise training was due, at least partially, to the reduction of adipose tissue mass and to the consequent reduced adipokine secretion in the EXE group. Notably, recent studies demonstrated that very low-calorie diet significantly reduced serum concentration and peripheral monocyte expression of proinflammatory markers and specific components of IGF-1 axis in adult nondiabetic [43] and diabetic [44] obese women. Accordingly, this study demonstrated that concurrent training increases fat-free mass along with muscle strength and endurance and is associated with an increase of IGF-1 plasma level and of its mRNA expression in PBMC. It is well known that ageing is associated not only with an increase in fat mass but also with sarcopenia and dynapenia [45]. In healthy subjects, the prevalence of sarcopenia is between 5% and 13% in the 60–70 yrs age range, increasing up to 50% in the next ten years [46]; these percentages are higher in subjects suffering from T2D [47]. This scenario highlights an important role for concurrent exercise as an effective strategy to counteract age-related sarcopenia. Exercised patients of the present study, in fact, improved muscular fitness and increase fat-free mass and IGF-1 level. The increase of plasma IGF-1 found in the present study is particularly important since low systemic IGF-1 levels have been linked, especially in elderly people, to the decline of skeletal muscle mass and function in T2D patients [48–50]. It is well established that insulin and IGF-1 signaling affect not only glucose uptake and metabolism but also muscle growth and protein turnover [48]. Indeed, IGF-1 treatment induces muscle hypertrophy and protects against age-related atrophy [48]. The IGF-1 pathway may also play a role in exercise-induced strength increases [51]. For example, after a 13-week resistance training intervention, circulating IGF-1 increased by approximately 20% in healthy, sedentary subjects [51], which is in line with the 16% increase found in the present study. Interestingly, we also found a modulation of *IGF-1* and *IGFBP-3* mRNAs in mononuclear cells after the exercise training. Recent studies demonstrated that monocyte/macrophage-derived IGF-1 is the major extrahepatic source of IGF-1 and macrophage-derived IGF-1 plays a significant role in coordinating muscle regeneration and inflammation following injury [52, 53]. These findings indicate that increased circulating IGF-1 and its PBMC mRNA expression may be at least partially responsible for fat-free mass and strength increases that occur as a result of concurrent training. However, further studies will be required to confirm this hypothesis since gain in strength and muscle mass may be brought by a number of other factors as well, including muscle IGF-1, testosterone, insulin, and neural adaptation [48, 51].

The results obtained from the plasma proteome support the anti-inflammatory effect of exercise training and its essential role to prevent long-term complications of diabetes. Particularly, of the 9 most differentially regulated spots found, 2 correspond to fibrinogen, which decreased after the training. Fibrinogen is the most important protein involved in the clotting process and also a potent proinflammatory mediator [54]. The training intervention also reduced the K light chain of immunoglobulin. In T2D patients, a significant increase in blood and urine-free light chains can be observed before the onset of overt renal impairment, suggesting that the excretion of free light chains may be a marker of susceptibility to diabetic nephropathy [55, 56]. The proteomic analysis also showed an increase of ApoA1 and ApoE after the training program. Other authors have reported a downregulation of apolipoproteins in poorly controlled diabetes [57], and serum apolipoprotein levels have been associated with diabetic retinopathy incidence and severity [58]. Hence, the increase of ApoA1 and ApoE in the EXE group may have a positive effect against the development of retinopathy, which is the leading cause of morbidity and disability in older diabetic patients [59]. Taken together, data of the present study indicate that the anti-inflammatory effects of exercise training are not only important in the pathogenesis of T2D but also, and perhaps even more importantly, in breaking the vicious cycle that leads to common diabetic complications, such as retinopathy, nephropathy, neuropathy and, above all, macrovascular diseases.

Despite the numerous benefits of exercise training on body composition and systemic inflammation, there was no statistical change in HbA1c between the control and exercise group. Glycemic control, based on measurement of HbA1c, has been the main outcome of meta-analyses assessing the efficacy of exercise training in T2D patients [60]. In our study, we observed a 0.4% reduction of HbA1c in the exercise group (7.1 ± 0.8 to 6.7 ± 0.7%) after training. The post intervention change in HbA1c was not significantly different between the exercise and control group (*p* = 0.236), which is primarily attributable to the 0.3% average reduction of HbA1c levels in the controls. However, it is important to highlight that the mean baseline value of HbA1c was close to the recommended value of 7% in both groups at baseline, and further improved after the intervention, hence patients had a relatively good glycemic control. We guess that the nonsignificant change in HbA1c can likely be explained by the relatively short duration of the training intervention (16 weeks), which may not have allowed the full effect of the intervention on HbA1c to occur, as suggested by Eves and Plotnikoff [61]. Since we did not perform a complete analysis of the various aspects of glycemic control (e.g., we did not measure residual beta-cell function), we are aware that we probably missed the possibility to highlight improvements in glycemic control independently from changes in HbA1c, which is the main limitation of the present study. The second limitation of the present study is that diet was not systematically controlled for. Nevertheless, since the participants of both groups underwent the same drug regimen and diet recommendation, under the supervision of an endocrinologist and a nutritionist, we are confident that the greater improvements of the EXE group compared to the CON group are mainly attributable to training and not to a possible change in dietary habits. This hypothesis is supported by the significantly higher post training improvement of aerobic and muscular fitness yielded by the EXE group compared to the CON group.

Self-directed long-term exercise programs have shown to be effective in improving health outcomes when T2D patients become more independent and self-motivated [62]. However, since dose and intensity of exercise need to be met in order to trigger the health-enhancing effects of exercise, the supervision of the training sessions becomes very important when short-term exercise programs are employed and/or patients are not yet independent. Therefore, the studies that did not reveal any significant anti-inflammatory effect of physical activity or exercise on inflammation could have been biased by the absence of strict exercise supervision. In the present study, all the training sessions were supervised by exercise specialists and tailored on each patient according to the results of the fitness assessments, which is a strength of the study.

## 5. Conclusions

This study demonstrated that 16 weeks of concurrent aerobic and resistance training, in addition to improving cardiorespiratory fitness, muscular fitness, and body composition, has anti-inflammatory effects, as shown by a decrease of systemic inflammation and PBMC activation in late middle-aged T2D patients.

The findings of the present study highlight the importance of implementing structured and supervised exercise training in T2D management, particularly in the short and medium-term, in order to meet the dose and intensity of exercise that trigger the reduction of the inflammatory environment associated with this age-related chronic disease.

## Supplementary Material

Table S1. Primers used in real-time RT-PCR quantification.

## Figures and Tables

**Table 1:** Patients anthropometrics, body composition, fitness assessment scores, and clinical parameters before and after the intervention.

Variable	Control group (CON)			Exercise group (EXE)			EXE versus CON
	Baseline	16 weeks	% change	Baseline	16 weeks	% change	*p*
Age (yr)	60 ± 6.8	/	/	57 ± 9.1	/	/	/
Duration of diabetes (yr)	7.8 ± 5.2	/	/	10.1 ± 7.3	/	/	/
Body weight (kg)	85.6 ± 10.2	86.6 ± 9.9	0.5 (−1.2, 2.1)	85.6 ± 8.2	83.5 ± 9.8	−2.6 (−4.2, −1.1)	**0.011**
BMI (kg/m^2^)	29.0 ± 3.8	29.1 ± 3.6	0.4 (−1.4, 2.2)	28.3 ± 1.5	27.7 ± 2.1	−2.5 (−4.2, −0.8)	**0.029**
Waist circumference (cm)	102.1 ± 11.1	102.4 ± 11.5	0.3 (−0.8, 1.4)	101.2 ± 7.4	98.6 ± 6.4	−2.4 (−3.5, −1.4)	**0.002**
Hip circumference (cm)	102.4 ± 6.2	102.9 ± 5.9	0.5 (−0.7, 1.6)	103.5 ± 5.0	101.4 ± 4.9	−2.0 (−3.1, −1.0)	**0.004**
Fat mass (%)	29.7 ± 2.0	30.3 ± 1.2	2.0 (−1.3, 5.4)	29.5 ± 5.9	27.5 ± 6.3	−7.4 (−10.7, −4.0)	**0.001**
Fat-free mass (%)	70.3 ± 6.2	69.7 ± 5.2	−0.5 (−2.6, 1.7)	70.5 ± 5.9	72.5 ± 6.3	2.8 (2.0, 3.5)	**0.001**
VO_2max_ (mL/kg/min)	37.2 ± 7.7	37.0 ± 11.5	−2.3 (−11.0, 6.4)	33.3 ± 3.8	38.9 ± 5.3	18.1 (9.9, 26.2)	**0.003**
1-RM leg press (kg)	182.6 ± 31.7	188.1 ± 52.4	1.6 (−12.6, 15.7)	168.9 ± 30.5	199.7 ± 44.3	19.4 (6.1, 32.7)	**0.043**
m-RM leg press (reps)	26.4 ± 9.7	29.1 ± 6.0	27.8 (−19.8, 75.4)	27.1 ± 6.4	47.6 ± 19.0	86.9 (42.0, 131.8)	**0.043**
1-RM chest press (kg)	55.1 ± 6.9	55.9 ± 6.2	0.4 (−0.9, 1.6)	59.6 ± 13.5	68.6 ± 13.6	3.7 (2.6, 4.9)	**0.001**
m-RM chest press (reps)	31.8 ± 8.5	31.6 ± 8.3	−4.2 (−33.1, 24.8)	24.5 ± 6.0	35.9 ± 14.3	45.6 (21.7, 69.5)	**0.020**
1-RM lat pull-down (kg)	74.1 ± 12.1	75.3 ± 7.7	4.2 (−6.3, 14.6)	72.6 ± 7.6	80.2 ± 13.2	9.6 (−0.1, 19.3)	**0.040**
m-RM lat pull-down (reps)	46.1 ± 9.9	46.3 ± 10.5	1.4 (−12.7, 15.4)	38.3 ± 4.8	46.2 ± 4.5	20.3 (8.4, 32.3)	**0.040**
Systolic BP (mmHg)	130.6 ± 12.4	128.1 ± 10.7	−0.4 (−7.0, 6.2)	126.6 ± 12.7	116.6 ± 12.9	−8.5 (−14.6, −2.3)	**0.048**
Diastolic BP (mmHg)	80.6 ± 3.2	82.5 ± 5.3	1.8 (−2.3, 5.8)	81.9 ± 6.3	76.6 ± 3.5	−5.5 (−9.3, −1.7)	**0.014**
Fasting blood glucose (mg/dL)	129.4 ± 36.0	164.0 ± 59.5	58.9 (−0.71, 18.6)	128.3 ± 27.5	123.6 ± 24.3	−1.5 (−57.8, 54.7)	0.136
HbA1c (%)	7.2 ± 1.1	6.9 ± 0.8	−2.6 (−6.4, 1.3)	7.1 ± 0.8	6.7 ± 0.7	−5.6 (−9.2, −2.0)	0.236
Total cholesterol (mg/dL)	183.8 ± 30.3	190.4 ± 16.6	5.0 (−4.6, 14.9)	185.1 ± 21.2	169.1 ± 34.2	−8.3 (−17.6, 1.0)	**0.049**
LDL cholesterol (mg/dL)	123.3 ± 22.1	124.1 ± 12.3	7.0 (−6.6, 20.5)	107.9 ± 16.3	105.6 ± 26.7	−4.7 (−17.4, 8.0)	0.216
HDL cholesterol (mg/dL)	41.6 ± 14.2	42.7 ± 10.8	4.8 (−3.3, 12.9)	42.1 ± 8.6	38.9 ± 4.3	−4.5 (−13.1, 4.2)	0.115
Creatininemia (mg/dL)	0.9 ± 0.1	0.9 ± 0.1	−4.7 (−8.8, −06)	1.0 ± 0.2	0.9 ± 0.2	−4.2 (−8.1, −0.3)	0.844

BMI: body max index; VO_2max_: maximal oxygen consumption; 1-RM: 1 repetition maximum; m-RM: multiple RM; BP: blood pressure; HbA1c: glycated hemoglobin; *p*: level of statistical significance (bold when lower than 0.05). Absolute values are expressed as means ± SD; percentage changes are presented as means and 95% confidence interval.

**Table 2:** Concentrations of circulating adipokines, cytokines, chomokines, and hs-CRP before and after the intervention.

Variable	Control group (CON)			Exercise group (EXE)			EXE versus CON
	Baseline	16 weeks	% change	Baseline	16 weeks	% change	*p*
Leptin (ng/mL)	5.7 ± 2.7	5.2 ± 2.5	16.7 (−7.1, 40.6)	5.4 ± 1.8	3.6 ± 1.5	−33.9 (−59.3, −8.4)	**0.006**
Adiponectin (*μ*g/mL)	2.9 ± 1.0	2.8 ± 1.6	−3.2 (−20.5, 14.1)	2.3 ± 0.9	2.2 ± 1.0	−4.8 (−23.4, 13.7)	0.897
RBP4 (*μ*g/mL)	27.7 ± 4.0	26.7 ± 5.1	−5.6 (−12.1, 1.0)	30.6 ± 9.7	22.0 ± 4.4	−21.3 (−28.3, −14.3)	**0.003**
IL-6 (pg/mL)	1.1 ± 0.5	1.1 ± 0.5	2.0 (−11.5, 15.6)	1.3 ± 0.5	0.8 ± 0.4	−25.3 (−39.8, −10.8)	**0.009**
MCP-1 (pg/mL)	200.4 ± 41.6	217.8 ± 51.9	11.3 (−1.2, 23.8)	194.1 ± 42.1	166.4 ± 59.0	−15.3 (−28.7, −1.9)	**0.006**
TNF-*α* (pg/mL)	2.0 ± 0.2	2.1 ± 0.2	2.0 (−5.0, 8.9)	2.1 ± 0.5	1.6 ± 0.4	−19.8 (−25.4, −14.1)	**0.001**
hs-CRP (mg/L)	1.0 ± 0.8	0.9 ± 0.5	−12.5 (−81.5, 56.4)	1.6 ± 1.2	1.2 ± 0.5	61.3 (5.3, 117.2)	0.106
IGF-1 (ng/mL)	58.0 ± 11.3	61.3 ± 8.0	7.3 (1.0, 13.6)	57.1 ± 11.4	66.4 ± 14.9	16.4 (11.3, 21.5)	**0.029**
IGFBP-3 (*μ*g/mL)	1.9 ± 0.1	1.9 ± 0.2	2.4 (−1.8, 6.5)	1.8 ± 0.3	1.9 ± 0.4	3.5 (−1.0, 8.0)	0.705

RBP4: retinol-binding protein 4; IL-6: interleukin-6; MCP-1: monocyte chemotactic protein-1; TNF-*α*: tumor necrosis factor-alpha; hs-CRP: high sensitivity C-reactive protein; IGF-1: insulin-like growth factor-1; IGFBP-3: insulin-like growth factor binding protein-3; *p*: level of statistical significance (bold when lower than 0.05). Absolute values are expressed as means ± SD; percentage changes are presented as means and 95% confidence interval.

**Table 3:** Plasma protein differentially expressed after the intervention.

Spot	Protein name	NCBI ID
1	Ig kappa chain C region	IGKC_HUMAN
2	Ig kappa chain V-II region	KV206_HUMAN
3	Ig kappa chain C region	IGKC_HUMAN
4	Fibrinogen beta chain	FIBB_HUMAN
5	Fibrinogen beta chain	FIBB_HUMAN
6	Retinol-binding protein 4	RETBP_HUMAN
7	Apolipoprotein A1	APOA1_HUMAN
8	Apolipoprotein E	APOE_HUMAN
9	Transthyretin	TTR_HUMAN
