# Supplementary material for: Concurrent Aerobic and Resistance Training Has Anti-Inflammatory Effects and Increases Both Plasma and Leukocyte Levels of IGF-1 in Late Middle-Aged Type 2 Diabetic Patients
Source: Oxid Med Cell Longev. 2017 Jun 21;2017:3937842. doi: 10.1155/2017/3937842 (PMC5497609; doi:10.1155/2017/3937842)
Supplement: Supplementary file 1 — Table S1. Primers used in real-time RT-PCR quantification. [file 3937842.f1.doc]

| **Table S1.** Primers used in real-time RT-PCR quantification. | | |
| --- | --- | --- |
| Gene | Primer forward (5’-3’) | Primer reverse (5’-3’) |
| *IL-6* | GGTACATCCTCGACGGCATCT | GTGCCTCTTTGCTGCTTTCAC |
| *TNF-α* | CTGCTGCACTTTGGAGTGAT | CGGGGTTCGAGAAGATGAT |
| *IGF-1* | TCTCTTCTACCTGGCGCTGT | AAGCAGCACTCATCCACGAT |
| *IGFBP-3* | GGCCATGACTGAGGAAAGGA | CCTGACTTTGCCAGACCTTCT |
| *GAPDH* | AAATCAAGTGGGGCGATGCT | TGCTGATGATCTTGAGGCTG |
| Note: *IL-6*, interleukin-6; *TNF-α*, tumor necrosis factor-alpha; *IGF-1*, insulin-like growth factor-1; *IGFBP*, insulin-like growth factor binding protein-3: *GAPDH*, gliceraldeyde-3-phosphate dehydrogenase. | | |
